# Purple Urine Bag Syndrome in Two Elderly Men with Urinary Tract Infection

**DOI:** 10.1155/2015/746981

**Published:** 2015-08-17

**Authors:** Jan Van Keer, Daan Detroyer, Bert Bammens

**Affiliations:** ^1^Department of Nephrology, Dialysis and Renal Transplantation, University Hospitals Leuven, Herestraat 49, 3000 Leuven, Belgium; ^2^Department of Microbiology and Immunology, KU Leuven, Minderbroedersstraat 10, 3000 Leuven, Belgium

## Abstract

Purple urine bag syndrome is a rare condition in which purple discoloration of urine inside its collection bag occurs. We describe two illustrative cases. The first patient is an 81-year-old man who was hospitalized for a newly diagnosed lymphoma with acute obstructive renal failure for which a nephrostomy procedure was performed. During the hospitalization, a sudden purple discoloration of the suprapubic catheter urine was noted, while the nephrostomy urine had a normal color. Urine culture from the suprapubic catheter was positive for *Pseudomonas aeruginosa* and *Enterococcus faecalis*; urine from the nephrostomy was sterile. The second case is an 80-year-old man who was admitted for heart failure with cardiorenal dilemma and who was started on intermittent hemodialysis. There was a sudden purple discoloration of the urine in the collection bag from his indwelling catheter. He was diagnosed with an *E. coli* urinary infection and treated with amoxicillin and removal of the indwelling catheter. These two cases illustrate the typical characteristics of purple urine bag syndrome.

## 1. Introduction

Purple urine bag syndrome is a rare condition characterized by a purple discoloration of urine inside the urine collection bag [1]. It is mostly seen in patients with chronic urinary catheterization, constipation, and urinary tract infection [2]. The purple color is thought to be caused by bacterial metabolization of dietary tryptophan into indigo and indirubin inside the urinary catheter system [3]. Here, we describe two illustrative cases.

## 2. Case 1

An 81-year-old man was admitted to the nephrology ward because of anorexia, weight loss, and acute on chronic renal failure. His past medical history was remarkable for atrophy of the left kidney, myocardial infarction, follicular type non-Hodgkin lymphoma treated with CHVmP/BV chemotherapy (19 years prior to the present admission), and prostate cancer treated with androgen deprivation (6 years prior to the present admission). The patient had a permanent suprapubic catheter that was changed at 6-weekly intervals. On admission, he was found to have extensive supra- and infradiaphragmatic lymphadenopathy, with encasement of the right ureter and hydronephrosis of the right kidney. An urgent nephrostomy had been performed. Biopsy of a palpable cervical lymph node later revealed diffuse large B-cell lymphoma.

**Figure 1:**
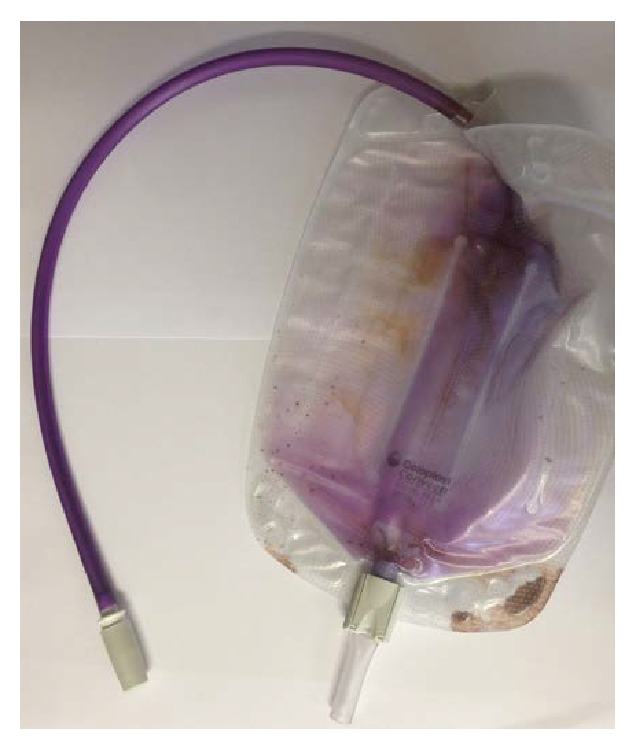
Purple discoloration of urine bag and tubing coming from suprapubic catheter. Note that the nephrostomy urine bag (not shown) had a normal color.

During the hospitalization, a sudden purple discoloration of the suprapubic urine collection bag was noted (see Figure 1). The nephrostomy urine had a normal color. The patient had been constipated during the preceding days, for which he had been treated with macrogol laxatives. His other medications had not been changed and included enoxaparin, aspirin, bisoprolol, amlodipine, rosuvastatin, sertraline, and three-monthly injection of goserelin. The patient had no other complaints.

Vital parameters were normal. Physical exam showed right cervical lymphadenopathy and the presence of a nephrostomy with normal colored urine and a suprapubic catheter with obvious purple color of urine bag and tubing. Laboratory evaluation revealed renal insufficiency with creatinine of 1.49 mg/dL and blood urea nitrogen of 25 mg/dL (creatinine at admission, before the nephrostomy procedure, had been 6.36 mg/dL). Hemogram showed a mild normochromic normocytic anemia with hemoglobin of 10.5 g/dL; white blood cell differential count and platelets were normal. Liver function tests were within normal limits. Lactate dehydrogenase was elevated (505 U/L): this was attributed to the diffuse large B-cell lymphoma. The level of CRP was 25 mg/L (reference value < 5 mg/L). Analysis of the suprapubic catheter urine showed pyuria and trace hematuria. Urinary pH was 9.0. Urine culture came back positive for* Pseudomonas aeruginosa* and* Enterococcus faecalis*. Analysis of the nephrostomy catheter urine showed trace hematuria, with a pH of 6.0 and a negative culture. As the nephrostomy urine was sterile and the patient had no signs of urinary infection, no antibiotics were given. The purple discoloration gradually disappeared during the following week. At discharge, kidney function had improved back to baseline with creatinine of 1.20 mg/dL. The patient was subsequently treated with R-CVP chemotherapy, with remission after 6 cycles.

## 3. Case 2

An 80-year-old man was hospitalized because of heart failure with cardiorenal dilemma. His past medical history included coronary bypass surgery, ischemic cardiomyopathy, chronic kidney disease, chronic obstructive pulmonary disease, prostate cancer treated with total androgen deprivation, and type 2 diabetes, treated with diet only. His medication included pantoprazole, enoxaparin, aspirin, bumetanide, carvedilol, atorvastatin, bicalutamide, darbepoetin alfa, macrogol laxatives, and fluticasone, salmeterol, and tiotropium inhalation therapy. He had been admitted with pulmonary edema, for which continuous venovenous hemofiltration (CVVH) was started. Afterwards, he was transferred to the nephrology ward for continuation of chronic intermittent hemodialysis. He had a residual diuresis of 600 mL per 24 hours. An indwelling urinary catheter had been placed on the first day of hospitalization to monitor urinary output.

**Figure 2:**
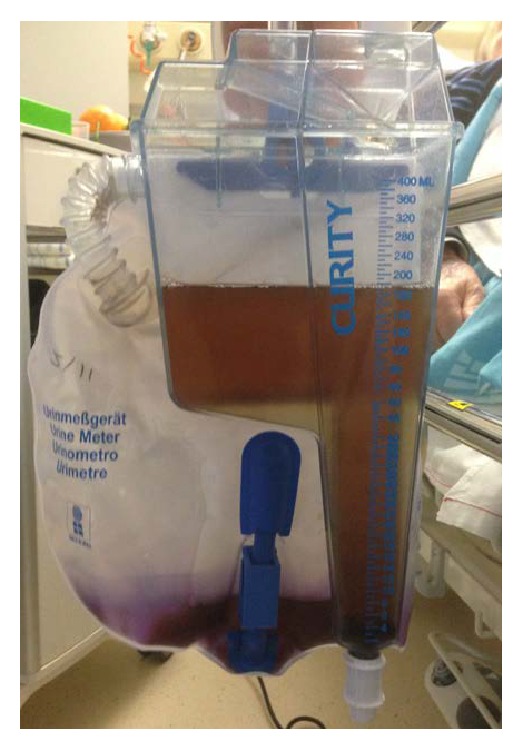
Purple color of the urine bag. Note that the color of the urine in the collection device before entering the bag is normal.

On the 13th day of hospitalization, the urine inside the urine bag suddenly turned purple (see Figure 2). The patient had a burning sensation in the lower abdomen. Vital parameters were unremarkable. Physical examination showed bilateral basal crepitations on auscultation, systolic cardiac murmur, mild peripheral edema, and indwelling urinary catheter with remarkable purple color of the collecting bag, while the urine in the tubing before the bag had a normal color. Laboratory assessment showed anemia (hemoglobin 8.9 g/dL) with mild macrocytosis, normal leukocyte count and differentiation, and platelets of 115 × 109/L. Creatinine before dialysis was 3.11 mg/dL and blood urea nitrogen was 48 mg/dL. Liver function tests were normal. CRP was 23 mg/L (reference value < 5 mg/L). Urine analysis showed pyuria and trace hematuria; urinary pH was 8.5. Culture was positive for* Escherichia coli*. The indwelling catheter was removed. The patient was treated with amoxicillin for 7 days. He was discharged in good health, with continuation of thrice-weekly hemodialysis. Shortly thereafter, the patient developed urinary obstruction for which a permanent suprapubic catheter was placed. The purple urine color never recurred.

## 4. Discussion

Purple urine bag syndrome is a rare condition that can seem alarming, but it is mostly benign. The syndrome was first described in 1978 [1]. Since then, less than 100 cases have been published. Most are institutionalized female patients with chronic indwelling urinary catheters. Purple urine bag syndrome is associated with urinary tract infection and with constipation [2].

The pathogenesis is controversial. According to the most popular hypothesis [3], dietary tryptophan is converted to indole by gut bacteria, which is further metabolized in the liver to indoxyl sulphate and then excreted in the urine. Constipation favors conversion of tryptophan to indole by gut bacteria.

**Figure 3:**
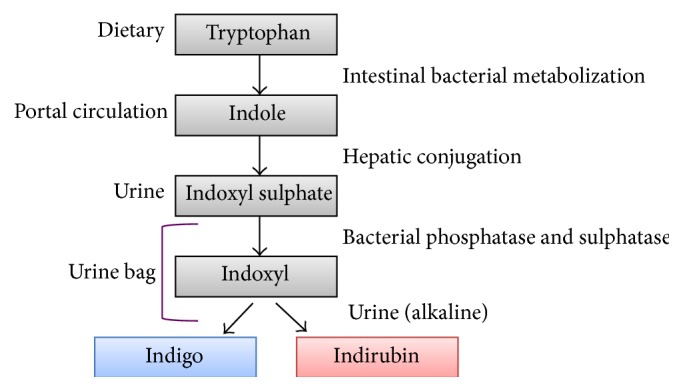
Pathogenesis of the purple urine bag syndrome (adapted from Hadano et al. [2]).

Once excreted, indoxyl sulphate can be processed by bacteria colonizing the urinary catheter to indoxyl, which is further converted to indigo (blue) and indirubin (red). These pigments result in a deep purple color in interaction with the plastic tubing (see Figure 3). The most commonly involved bacteria are* Providencia stuartii*,* Providencia rettgeri*,* Escherichia coli*,* Klebsiella pneumoniae*,* Proteus mirabilis*,* Morganella morganii*,* Pseudomonas aeruginosa*, and* Enterococcus* species [4]. These bacteria produce indoxyl phosphatase and sulphatase enzymes. Although alkaline urine is an important risk factor, purple urine bag syndrome has been described in acidic urine as well [5].

Note that, in our first patient, only the suprapubic urine bag (with* Pseudomonas* colonization) turned purple, whereas the (sterile) nephrostomy bag did not. The second case shows that the discoloration only occurred after contact with the plastic bag.

Purple urine bag syndrome is a sign of colonization of the urinary catheter system. Antibiotic therapy is only indicated in patients with symptomatic urinary infection. For asymptomatic patients, treatment of underlying risk factors (e.g., constipation) might suffice [6]. The mainstay of preventing purple urine bag syndrome is the avoidance of chronic catheterization by prompt removal of urinary catheters once they are no longer needed.
